# Docking and Activity of DNA Polymerase on Solid-State Nanopores

**DOI:** 10.1021/acssensors.2c00216

**Published:** 2022-05-10

**Authors:** Shiyu Li, Shuangshuang Zeng, Chenyu Wen, Zhen Zhang, Klas Hjort, Shi-Li Zhang

**Affiliations:** † Department of Electrical Engineering, Division of Solid-State Electronics, Uppsala University, SE-751 03 Uppsala, Sweden; ‡ Department of Material Science and Engineering, Division of Microsystem Technology, Uppsala University, SE-751 21 Uppsala, Sweden

**Keywords:** solid-state nanopore array, DNA polymerase, rolling circle amplification, truncated-pyramidal nanopore, label-free detection, hafnium oxide

## Abstract

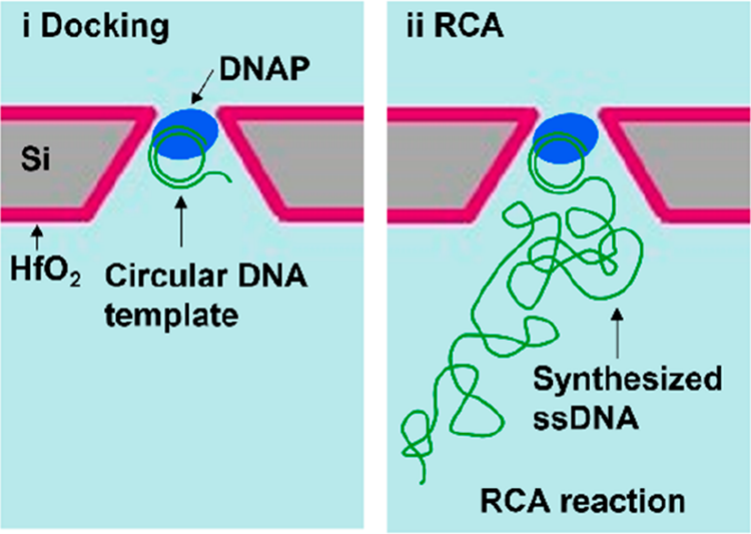

Integration of motor enzymes with biological nanopores has enabled commercial DNA sequencing technology; yet studies of the similar principle applying to solid-state nanopores are limited. Here, we demonstrate the real-life monitoring of phi29 DNA polymerase (DNAP) docking onto truncated-pyramidal nanopore (TPP) arrays through both electrical and optical readout. To achieve effective docking, atomic layer deposition of hafnium oxide is employed to reduce the narrowest pore opening size of original silicon (Si) TPPs to sub-10 nm. On a single TPP with pore opening size comparable to DNAP, ionic current measurements show that a polymerase–DNA complex can temporally dock onto the TPP with a certain docking orientation, while the majority become translocation events. On 5-by-5 TPP arrays, a label-free optical detection method using Ca^2+^ sensitive dye, are employed to detect the docking dynamics of DNAP. The results show that this label-free detection strategy is capable of accessing the docking events of DNAP on TPP arrays. Finally, we examine the activity of docked DNAP by performing on-site rolling circle amplification to synthesize single-stranded DNA (ssDNA), which serves as a proof-of-concept demonstration of utilizing this docking scheme for emerging nanopore sensing applications.

Nanopores have been recognized as an ultimate analytical tool with unique capabilities of sensing and manipulating single molecules.^1−3^ Starting with the initial goal for DNA sequencing, nanopore-based sensing has, to date, been explored in a broad range of applications such as fundamental biophysical studies,^4,5^ biological screening,^6^ and even protein sequencing.^7−9^ However, with the typical sensing principle of monitoring the ionic current blockade, the fast translocation of analyte has limited the extraction of detailed molecular information. To increase the dwell time or control the movement of the target analyte in the sensing region, various approaches have been developed, e.g., carrying anchored protein through the pore by the diffusion of fluid lipid bilayer^10,11^ and stalling molecules in the pore with optical^12,13^ or magnetic tweezers.^14^ Another prominent approach is to dock a molecular plug tethered or conjugated with target molecules onto the nanopore. With this approach, the analyte is stalled at the sensing region to be probed. In particular, this technique has enabled reading DNA sequences with biological pores^15^ where the docked molecular plug is a motor enzyme, such as DNA polymerase^15^ or helicase,^16^ to control the DNA movement.

However, in the case of solid-state nanopores, little has been explored on utilizing this docking-sensing scheme for potential biosensing applications. One of the concerns is that the docked enzyme may lose its activity due to undesirable docking orientations in a confined space or strong interactions between the enzyme and the pore surface.^17,18^ Such attempts on solid-state nanopores should ideally be pursued in a high-throughput manner, which allows for a thorough examination of both active and inactive enzyme docking. However, in the case of electrical detection, each pore in the sensor array needs to be monitored independently, which requires advanced microfluidics and sophisticated contact electrode design for multiplexed readout. On the other hand, common optical methods require labeling of the target analytes to enable simultaneous detection with multiple nanopores. This requirement makes the detection restrictive and susceptible to photobleaching and weak signals.^19^ To address these limitations, a label-free optical method was previously developed for monitoring ion flow through protein channels^20,21^ or solid-state nanopores.^22,23^ This method employs a Ca^2+^-sensitive fluorescent dye to monitor changes in Ca^2+^ concentration in the vicinity of an ion channel. With this method, enzyme labeling can be avoided for optical observation, as long as the enzyme is compatible with the Ca^2+^ concentration used in the detection. Hence, this method can be adapted for parallel detection of the enzyme docking behavior on solid-state nanopores and subsequent exploration of this sensing scheme for potential enzymatic applications.

In the present work, we characterize the docking behavior of biomolecular complexes of DNA polymerase (DNAP) bound with a DNA template onto silicon (Si) based truncated-pyramidal nanopores (TPPs). We choose phi29 DNAP as the studied enzyme by considering its natural binding with a single-stranded DNA (ssDNA) template and its extensive usage in DNA synthesis for various sensing applications.^24,25^ Silicon-based TPP arrays are characterized by a substantially lower photoluminescence under the experimental conditions than the more commonly used silicon nitride nanopores.^26^ In addition, we utilize atomic layer deposition (ALD) of hafnium oxide (HfO_2_) to shrink initially large Si TPPs down to sub-10 nm opening size. The resulting pore size is comparable with the dimension of phi29 DNAP, which is crucial in preventing DNAP from slipping through the nanopores under the electrophoretic force. Electrical measurement using a single TPP is carried out to investigate the docking dynamics of the DNAP-template complex. Optical characterization using a Ca^2+^-sensitive dye is employed to detect the docking of DNAP on arrays of 5-by-5 TPPs, as illustrated in Figure 1. A docked DNAP can block the Ca^2+^ flow, thereby weakening or even prohibiting the fluorescence signal, which is an ideal scheme to confirm the molecular docking behavior on nanopore arrays. Finally, the activity of docked DNAP on TPP arrays is further examined by performing rolling circle amplification (RCA) to observe the synthesized ssDNA.

**Figure 1:**
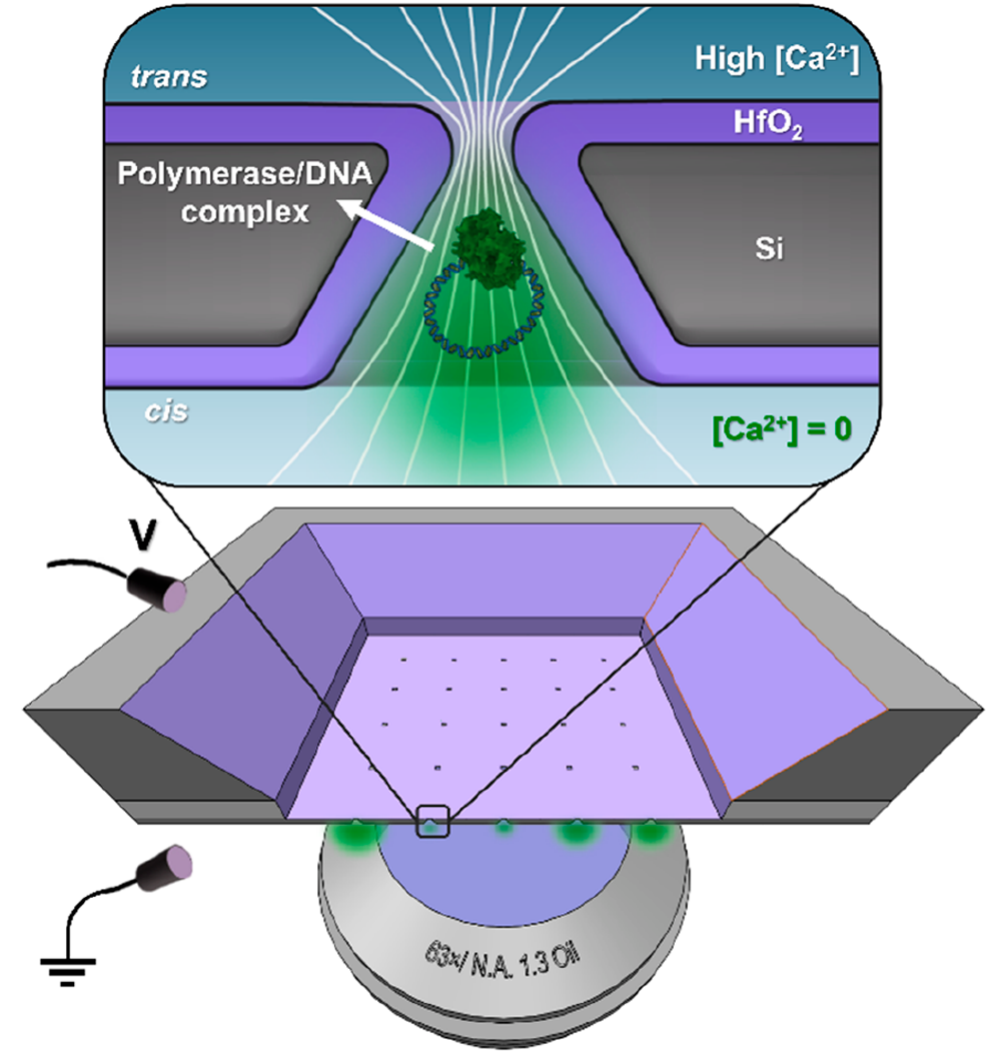
Schematic of the optical readout setup for label-free detection of polymerase-DNA complexes docking onto HfO_2_-coated TPP arrays. A voltage drop across the membrane drives Ca^2+^ ions to the *cis* side where they conjugate with Fluo-4 molecules, resulting in fluorescence signals of an open pore. The docking event of DNAP on TPPs can block the Ca^2+^ ion flow, thus resulting in a weakened or vanished signal. In the experiment, the *cis* side was always set to ground, while the *trans* side was biased to a specified voltage (positive, zero, or negative).

## Methods

### Fabrication of HfO_2_-Coated Si Nanopores

The TPPs were fabricated using a previously reported process,^27^ and a step-by-step process flow is provided in Figure S1 of Supporting Information. The fabrication process is described briefly here: it started with a 100 mm double-side-polished silicon on insulator (SOI) wafer with an 88-nm-thick single-crystal Si device layer on a buried oxide (BOX) layer. A 30 nm low-stress silicon nitride (SiN_*x*_) layer was deposited on both sides of the wafer via low-pressure chemical vapor deposition. This was followed by nanohole arrays patterned in the SiN_*x*_ layer using electron beam lithography and reactive ion etching (RIE). The substrate was opened with large cavities by deep RIE and KOH etching (80 °C) to stop on the BOX with the front Si device layer protected. In the next step, patterned nanoholes in SiN_*x*_ were transferred to the Si device layer with a second KOH etching (30 °C). The KOH etch is highly anisotropic with a much higher etch rate for the ⟨100⟩ crystal planes of Si than for the ⟨111⟩ ones, thereby leaving behind the naturally sloped sidewalls of etched nanopores in the Si device layer. After removal of BOX, TPPs in a free-standing Si membrane were formed. The SiN_*x*_ layer on the front side was removed by a further RIE process to reduce interfering fluorescence.^26^ Finally, an additional 10-nm-thick HfO_2_ layer was coated by means of ALD. Before measurements, the TPP chips were boiled in a freshly prepared piranha solution with H_2_SO_4_:H_2_O_2_ (3:1, v/v) for 30 min, followed by rinsing in deionized water and drying by nitrogen blow.

### Preparation of Polymerase–DNA Complex

To prepare circular and primed DNA templates, DNA ligation was performed. It included incubation of the 5′-phosphorylated template and primer at a 1:3 concentration ratio with T4 DNA ligase (2 U/μL) in a 1× T4 DNA ligase buffer solution (40 mM Tris-HCl, 10 mM MgCl_2_, 10 mM DTT, 5 mM ATP) at 37 °C for 1.5 h. It was followed by inactivation at 65 °C for 10 min. For binding polymerase to template, polymerase (8 nM) was incubated with the primer-bound template (1 nM) in a phi29 DNAP reaction buffer (33 mM Tris-acetate, 10 mM magnesium acetate, 66 mM potassium acetate, 1 mM DTT, 0.1% Tween-20) at 4 °C for 30 min. The sequences of the designed template and primer are as follows: template: 5′-p-GTTCTGTCATACAGTGAATGCGAGTCCGTCTAACTAGTGCTGGATGATCGTCCAAAGCGATCTGCGAGACCGTATAAGAGTGTCTA-3′, primer: 5′-AAAAAAAAAATATG-ACAGAACTAGACACTCTT-3′.

### Electrical Measurement

The ionic current measurement was implemented on a single TPP chip at a specified bias voltage applied to the *trans* side. The chip was sandwiched by a customized poly(methyl methacrylate) (PMMA) flow cell ceiled using O-rings. For DNAP docking detection, both chambers on the two sides of the TPP chip were filled with phi29 reaction buffer, with 8 nM DNAP-template complex. A pair of pseudo Ag/AgCl electrodes was used to apply a bias voltage between the two chambers. A patch clamp amplifier Axopatch 200B (Molecular Device Inc.) was used to measure ionic current changes in the system. Translocation and docking signals were acquired at 50 kHz sampling frequency with a 10 kHz four-pole Bessel low-pass filter. The whole setup was placed inside a Faraday cage in order to minimize coupling of external electromagnetic noise.

### Label-free Optical Detection System

The chip was mounted in a customized polyether ether ketone (PEEK) fluidic cell with the two pseudo Ag/AgCl electrodes to form a closed electrical loop between two chambers separated by the chip (see Figure S2 of Supporting Information). The bottom of the cell chamber was a 0.17-mm-thick silica glass allowing for a short working distance for optical observation. The *cis* chamber was filled with a 100 mM KCl solution with 10 mM Tris-EDTA, 10 mM EGTA buffered to pH 7.4, and 20 μM Ca^2+^ sensitive dye Fluo-4. For DNAP docking experiments, the *cis* chamber was filled with phi29 reaction buffer, with 8 nM DNAP-template complex, and 20 μM Fluo-4. The *trans* chamber was filled with a buffer containing 50 mM KCl and CaCl_2_. Fluorescence observation was conducted with a confocal laser scanning microscope (CLSM) (TCS SP8, Leica) equipped with an HC PL APO 63× glycerol objective (NA = 1.3). During the experiment, the *trans* side was set at +200 mV.

### Rolling Circle Amplification (RCA)

The buffer containing Ca^2+^ and Fluo-4 in the fluidic chambers were replaced by phi29 reaction buffer after multiple successful DNAP docking events were confirmed on a TPP array. A bias voltage was set at +200 mV during this DNAP docking step. Upon completion of the docking, the bias was switched off (i.e., the *trans* side was also set to ground). To fully remove the Ca^2+^, Fluo-4, and redundant DNAP, the chambers were gently rinsed by reaction buffer for about 2 min. To initiate RCA by the docked DNAP, the chambers were injected with reaction buffer containing 10 μM each of dATP, dCTP, dGTP, and dTTP (dNTPs). The RCA process was performed at room temperature for 90 min without bias. Finally, the ssDNA synthesized by the docked DNAP was labeled by the SYBR Gold nucleic acid gel stain.

## Results and Discussion

### Characterization of HfO_2_ Shrunk TPP

**Figure 2:**
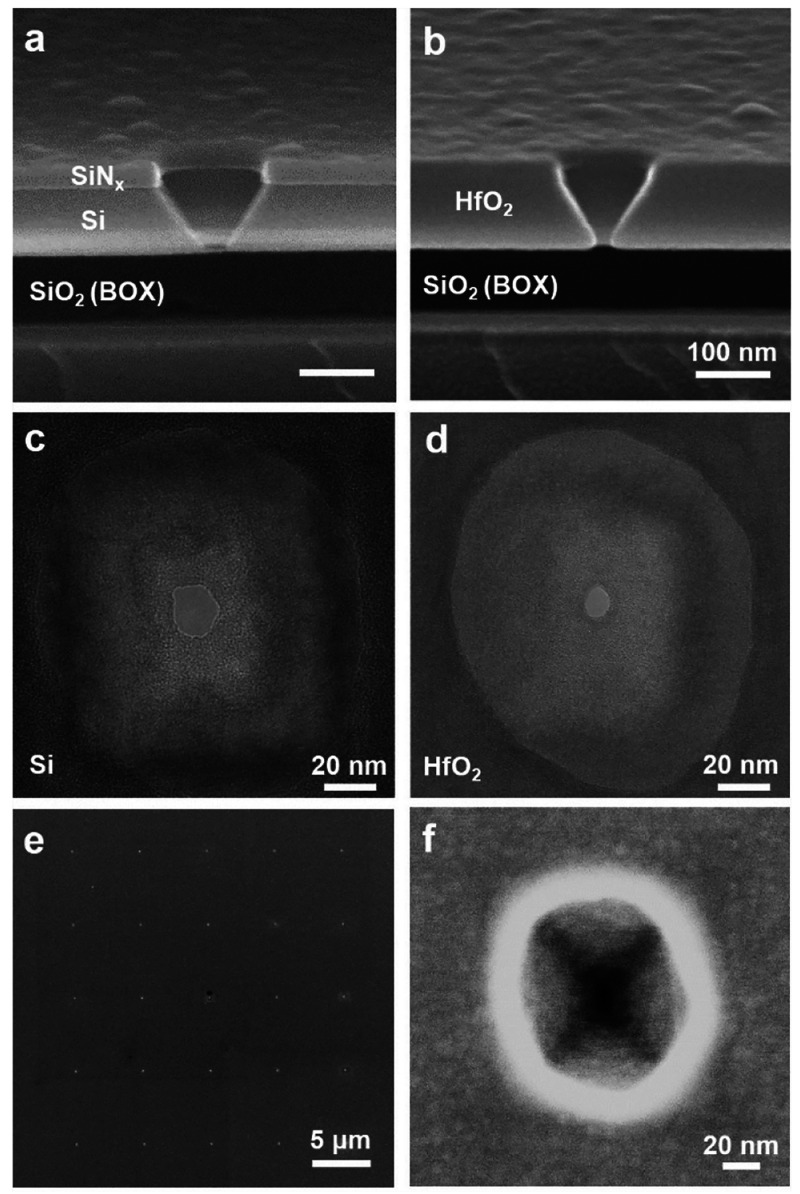
Characterization of the truncated-pyramidal shape nanopores. (a) Cross-sectional SEM image of an as-formed Si TPP with the SiN_*x*_ hardmask remained. (b) Cross-sectional SEM image of the TPP coated with a 10-nm-thick HfO_2_ after first removal of the SiN_*x*_ mask. (c) TEM image of an as-formed Si TPP with the SiN_*x*_ hardmask remained. (d) TEM image of a TPP after ALD coating of 10 nm HfO_2_ layer with a shrunk size of about 8 nm. (e) SEM image of a 5-by-5 HfO_2_ coated TPP array with a pore spacing of 6 μm. (f) Top-view SEM image of a single TPP with the short opening length of 8 nm.

To examine the geometry and size of as-fabricated and coated TPPs, both the original chip and the cross-sectioned sample were characterized by means of scanning electron microscopy (SEM) (see Figure S3 of Supporting Information for a dense nanopore array designed for facilitating cross-section images). The SEM image in Figure 2a shows a cross-sectional view of the initial Si pore in the truncated-pyramidal shape with the SiN_*x*_ mask layer remained, which results from the anisotropic etching of Si crystal in a KOH solution. After removal of the SiN_*x*_ layer, a 10-nm-thick HfO_2_ layer was deposited by means of ALD to shrink the pore opening below 10 nm for docking DNAP (shown in Figure 2b). Notably, the truncated pyramidal geometry is expected to be beneficial for hosting a docked DNAP because of a large contact area between the DNAP and the pore walls, especially when no external force is exerted on the DNAP. The geometry of the TPPs was further corroborated using transmission electron microscopy (TEM). The bright-field TEM images in Figure 2c,d are the top-view of a TPP before and after HfO_2_ coating, respectively; the latter shows the pore size shrunk to about 8 nm. A 5-by-5 HfO_2_ coated TPP array is depicted in Figure 2e with a pore spacing of 6 μm, which ensures an unambiguous identification of each pore under parallel optical observation. In the higher-magnification SEM image in Figure 2d, the short length of the rounded rectangular opening is about 8 nm. Even though a thicker HfO_2_ layer could be deposited to further shrink the pore size, we found that the HfO_2_ coating tended to bridge over and close the pores of smaller opening size (see Figure S4 of Supporting Information), which could arise from an unstable precursor gas flow in the tiny nanopore channel during the ALD process. In addition, the slight nonuniformity of the pore size across the TPP array could render further shrinkage to a total blockage of some pores, thereby leaving only a few pores in the open state for a high-throughput parallel detection. Given that the phi29 DNAP molecule is about 5–8 nm in size, a certain fraction of the TPP array with 8 nm and smaller pore size is expected to cause DNAP docking owing precisely to the nonuniformity nature of the pores. Another advantage of HfO_2_ coating is its excellent long-term stability under nanopore experimental conditions in electrolytes,^28^ which protects the Si TTP from pore size expansion.^28−30^

### Electrical Monitoring of DNAP Docking on a Single TPP

**Figure 3:**
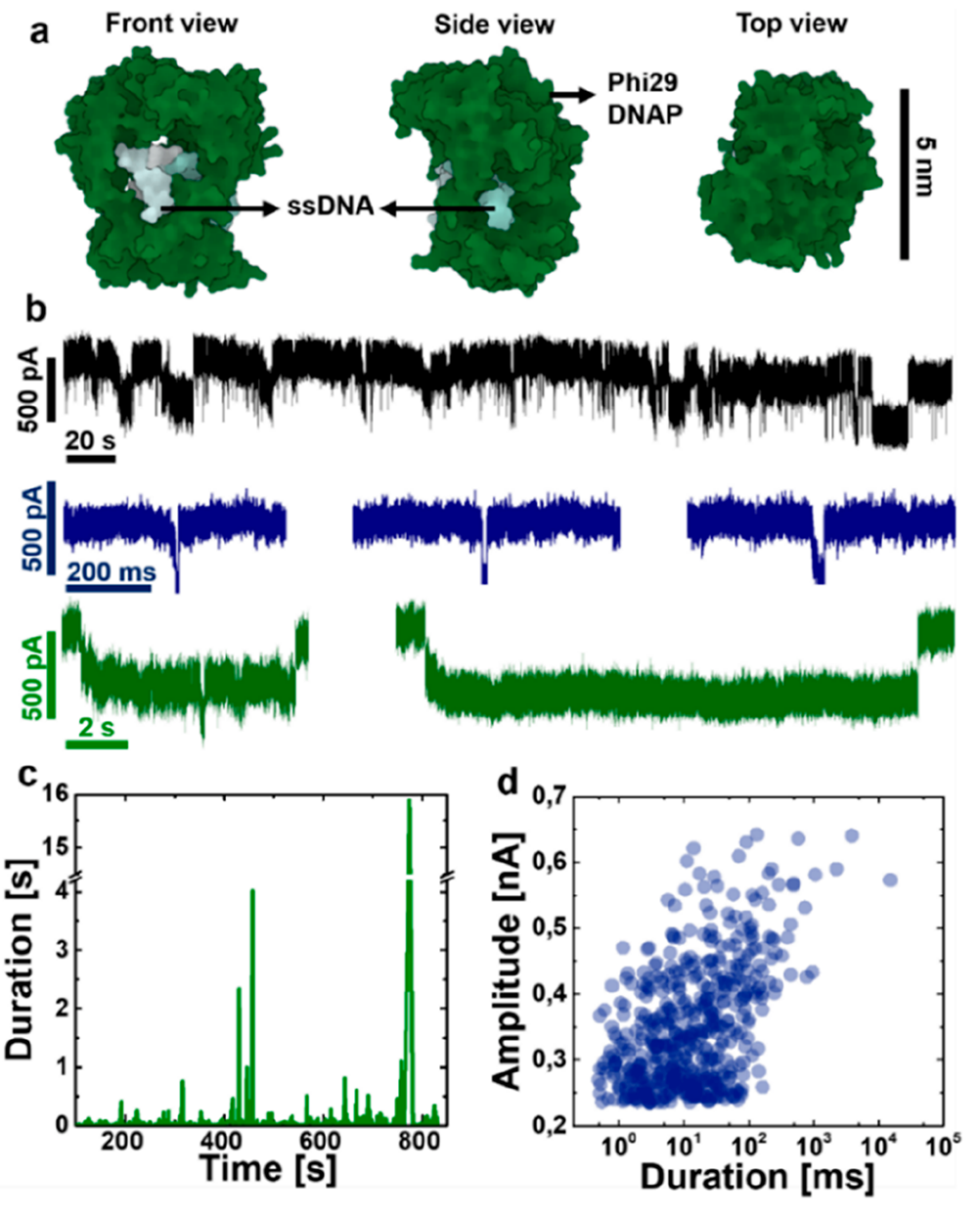
Electrical monitoring of DNAP docking on a single TPP: (a) Molecular surface structure of phi29 DNAP bound with an ssDNA molecule from different viewing angles. Images are obtained from Protein Data Bank (PDB); the PDB ID of this complex is 2PY5. (b) Typical ionic current traces of the phi29 DNAP bound with a circular DNA template at +200 mV passing/docking a single TPP: a continuous ionic current trace (black); example traces of translocation (blue) and docking (green). (c) Event duration vs time analyzed from the continuous ionic current traces shown in (b). The time coordinate represents the recording time of the measurement, where the origin indicates the beginning moment of the recording. (d) Amplitude and duration of detected events collected from the DNAP docking measurement at +200 mV.

To investigate the dynamics of DNAP captured by a TPP with a comparable size, DNAP docking on a single TPP with its opening size of about 8 nm (calculated from pore conductance measurement) was first electrically characterized. This pore size is similar to the average one of the TPP array used for the optical detection. The phi29 DNAP, whose shape and dimensions are displayed in Figure 3a, is bound to an 86-nt circular ssDNA template hybridized with a 32-nt primer ssDNA. The negatively charged DNAP-template complex can be electrophoretically driven to the TPP. At +200 mV bias voltage, clear translocation and long-duration docking events of the DNAP-template complex can be identified by monitoring how the ionic current traces vary with time. Typical examples of such traces are shown in Figure 3b with the blue ones for translocation and the green ones for docking. It shows in Figure 3c the event duration versus time plot from the continuous ionic current trace. Since the pore size of the TPP arrays used is not sufficiently small to totally prevent the translocation of the DNAP–template complex, the majority of detected events are related to translocations. This observation is further confirmed by analyzing the distribution of the event amplitude and duration in Figure 3d. A large population of the detected events has a duration time in the range of 1–100 ms, while a smaller population is beyond 100 ms with a few events lasting for seconds. The latter is attributed to the occasional docking of the DNAP–template complex on the TPP likely related to certain configurations or orientations in the pores. Protein translocation in solid-state nanopores is often characterized by a speed exceeding the electrical measurement bandwidth.^31−33^ Notably, the mean translocation time is reported to be about 1 μs for proteins with a range of molecular weights.^34^ To increase the dwell time of protein translocation, enhancing protein–pore interactions^35^ and regulating the electroosmotic flow (EOF) as a stalling force^26^ have been demonstrated to be effective. In this study, the observation of prolonged translocation events with a dwell time beyond 1 ms is likely due to the electrostatic attraction between the negatively charged DNA template and the slightly positively charged HfO_2_ pore sidewalls,^26^ as HfO_2_ has a point-of-zero-charge value about 7–8 and the buffer pH used here is about 7.4. In addition, the comparable size between the pores and the DNAP as well as the truncated-pyramidal geometry of the nanopore structure favor the docked DNAP to be totally confined inside the TPP. This configuration will provide a large contact area between the DNAP and the TPP sidewalls, which can further enhance the protein–pore interaction. The events with distinctive extraordinarily long dwell time beyond 1 s and a high blockade amplitude are regarded as successful in docking the DNAP–template complex onto the TPP instead of prolonged translocations. Such docking events are, however, unstable, because the electrophoretic force in collaboration with the electroosmotic force may force the complex to thread through the pore once its orientation relative to the pore favors such a movement. The dynamics of this movement has been investigated on docking the streptavidin–DNA complex on a 4 nm SiN_*x*_ pore,^36^ with the observation of a diversity of ionic current blockage due to the movement of the protein being docked at the nanopore entrance. Nevertheless, the observed occasional docking events with a duration length of several seconds allow us to proceed with the optical detection of DNAP docking on arrayed TPPs.

### Optical Detection of DNAP Docking on TPPs

**Figure 4:**
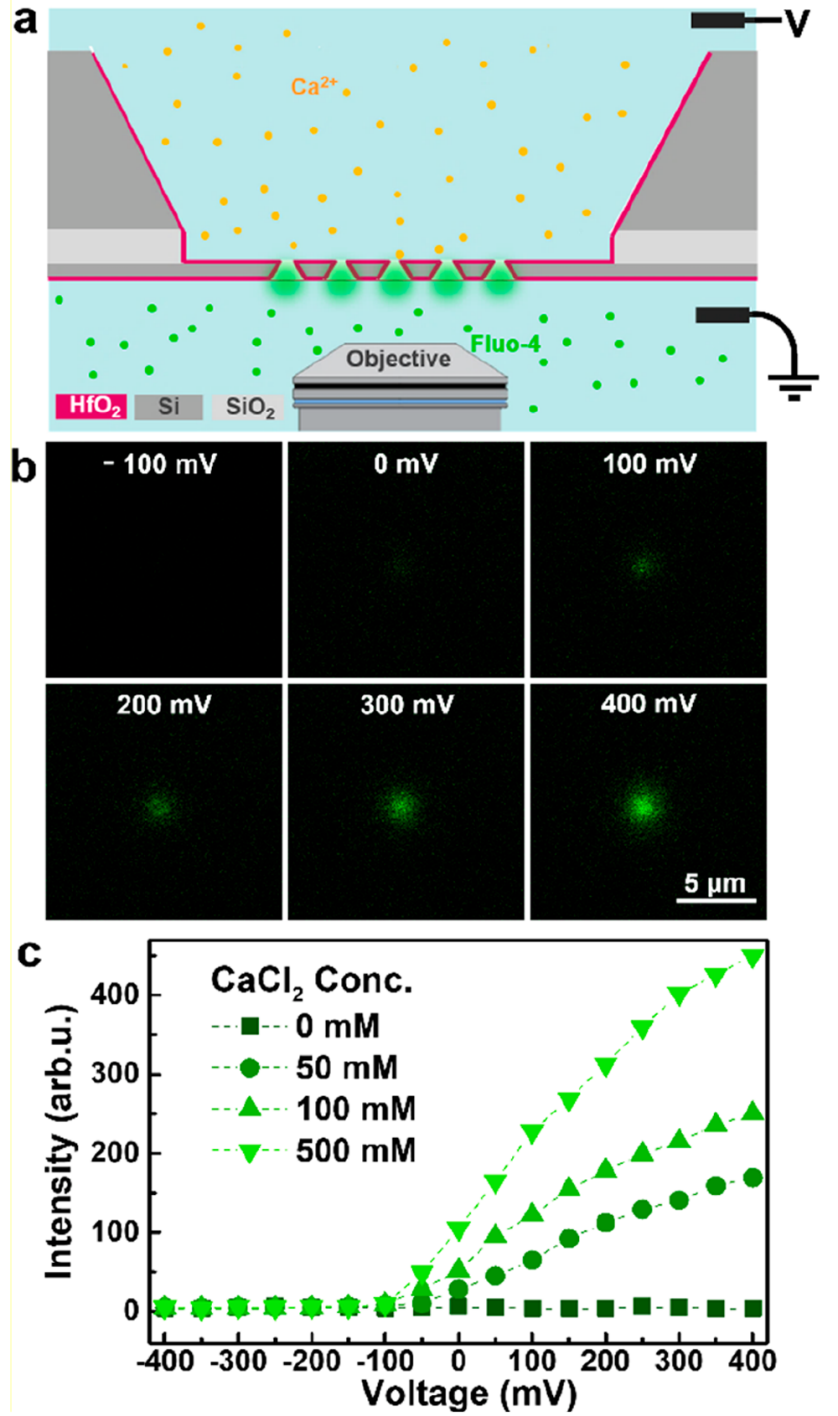
Ca^2+^ ion flow in arrayed TPPs inferred by means of optical detection: (a) Schematic of the label-free optical detection principle with Fluo-4 in the *cis* chamber and CaCl_2_ in the *trans* chamber. (b) Micrographs showing the fluorescence signals from a single TPP pore at different bias voltages with a bulk CaCl_2_ concentration of 100 mM. (c) Fluorescence intensity as a function of applied bias voltage at different bulk CaCl_2_ concentrations.

To optically detect the docking events of DNAP on the TPPs, the label-free optical detection method based on the Ca^2+^/Fluo-4 interaction was employed here. As illustrated in Figure 4a, Fluo-4 is used as a reporter molecule for Ca^2+^ in the vicinity of the nanopore, with Ca^2+^ ions and Fluo-4 molecules initially separated in the two chambers. As the Ca^2+^ ions are driven across the nanopores by a bias voltage, Fluo-4 molecules and Ca^2+^ ions can bind to form fluorescent dyes. Thus, the flow of Ca^2+^ ions can be inferred by monitoring the fluorescence signals on-site of the arrayed nanopores. To minimize the background noise, EGTA and EDTA were added to the chamber with Fluo-4 to chelate excessive Ca^2+^ ions. To confirm that the fluorescence intensity generated in the nanopore region was a positive function of the magnitude of Ca^2+^ flux through the pore, the fluorescence intensity was measured using confocal imaging with 20 μM Fluo-4 as a function of both Ca^2+^ concentration and applied bias voltage. As can be seen in the fluorescence images in Figure 4b, the intensity of the fluorescent spot on the TPP site is voltage-tunable. At a negative bias voltage of −100 mV, no fluorescent signal is observed due to the absence of Ca^2+^ flow to the *cis* chamber. At zero bias, a weak fluorescent signal is discernible at the pore due to the Brownian diffusive flow of Ca^2+^ ions. At positive bias, it is apparent that the fluorescence increases with voltage, which is the result of a higher level of Ca^2+^ flux (i.e., current) at higher voltage. The fluorescent intensity is seen in Figure 4c to rise above the background level at the onset voltage of −50 mV, and higher intensity values are measured at higher bulk CaCl_2_ concentration at the same voltage. Notably, the phi29 DNAP can only maintain its activity in buffer solution with Ca^2+^ concentration up to 70 mM as reported previously.^22^ Thus, to keep the DNAP active after docking and a sufficient optical intensity for detection, a Ca^2+^ concentration of 50 mM was chosen for further docking experiments.

**Figure 5:**
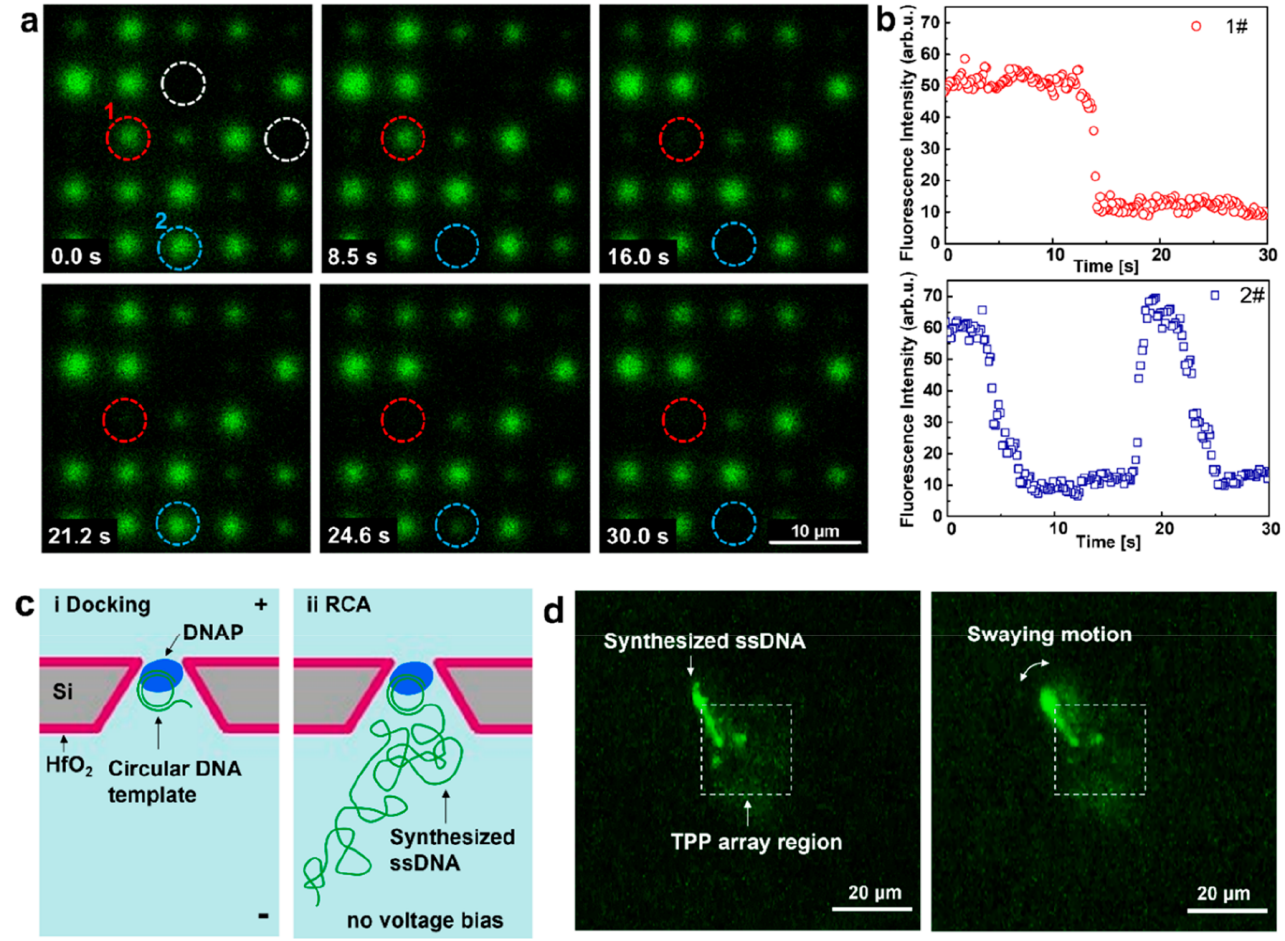
Optical detection of DNAP docking on arrayed TPPs and examination of DNAP activity by performing *in situ* RCA. (a) Set of fluorescence frames of docking DNAP–template complex onto TPPs in a 5-by-5 array with 20 μM Fluo-4 in the *cis* chamber and 50 mM CaCl_2_ in the *trans* chamber at +200 mV. The white dashed circles mark two pores without fluorescence signal, indicating that they were in a closed state after ALD coating. The red and blue dashed circles mark two pores showing docking behaviors. (b) Fluorescence modulations associated with docking the DNAP–template complex at +200 mV for the two marked pores in (a). (c) Schematic illustration of docking of the DNAP–template complex onto a TPP and subsequent *in situ* RCA to synthesize ssDNA after docking. (d) Fluorescence images of the labeled synthesized ssDNA after performing RCA for 90 min at room temperature. The long ssDNA can be clearly recognized with one end anchored on the TPP by the docked DNAP. A swaying motion of the tethered long ssDNA can also be seen from the real-time video.

To increase the efficacy in studying the docking behavior of DNAP on TPPs, experiments on 5-by-5 TPP arrays were carried out. Most of the TPPs in the array produced fluorescent signals at *t* = 0 s with 20 μM Fluo-4 in *cis* and 50 mM CaCl_2_ in *trans* at +200 mV, as seen in Figure 5a, indicating the presence of a Ca^2+^ ion flux. The difference in intensity is mainly due to the size nonuniformity with the TPP array. Two pores closed by ALD coating show no signal, which are marked with a white dashed circle. With a continuous recording for 30 s, obvious docking events can be identified at two pore positions marked with red and blue dashed circles by comparing with the respective state in the previous frames (Figure 5a). The profile of fluorescent intensity vs time at the two positions is extracted and shown in Figure 5b. The decreased fluorescence intensity lasting over seconds for pore 1# is clear evidence of a DNAP–template docking onto the TPP pore. In the case of pore 2#, it is also observed that the DNAP–template complex can pass through the pore after a docking duration of roughly 10 s, which is consistent with the electrical measurement of docking events on a single TPP. To achieve a more stable and efficient docking of phi29 DNAP, smaller TPP size below 5 nm is considered necessary. The expected prolonged translocation events as detected in the electrical measurement cannot be observed here, because the frame rate of the confocal imaging in our setup is limited to 7.9 Hz. The time interval of 126 ms between two consecutive frames is not able to detect translocation events. Nonetheless, the docking events can be clearly identified.

To further examine the activity of the docked DNAP, *in situ* RCA was performed to synthesize ssDNA after the successful docking events had been confirmed, as illustrated in Figure 5c. In detail, the bias voltage was switched off once multiple DNAP docking events were optically validated on a pore array. Then, the fluidic chambers were rinsed with phi29 reaction buffer to completely remove Ca^2+^ and Fluo-4. Subsequently, dNTPs (dATP, dCTP, dGTP, and dTTP) were added to the chambers to initiate the RCA process. After 90 min of reaction at room temperature, fluorescent DNA labeling dyes were added to the chambers. Immediately prior to the optical observation, a negative bias voltage of −50 mV was applied to stretch out the synthesized ssDNA for clear identification. As can be seen in Figure 5d, a clearly elongated ssDNA strand is obviously tethered to the pore membrane, which is considered evidence of the retained activity of DNAP on the TPP. Two relatively weak fluorescent spots are also observed in the TPP array region. They are likely to be short ssDNA synthesized by DNAP that became inactive during RCA, which may result from several different sources including undesired interaction with the pore sidewall surface and restricted local supply of dNTPs. The positions of the observed ssDNA molecules are correlated with the position of the TPPs (see Figure S5 of Supporting Information), which further validates that the ssDNA molecules are synthesized by the docked DNAP. Assuming a replication rate of phi29 DNAP of 1400–1500 bases/min,^37^ it results in about 135 000 nucleotides in the synthesized ssDNA, which is about 88 μm in its contour length. The observed long ssDNA is highly entangled due to its mechanical flexibility and emits a strong fluorescence signal. In addition, the swaying motion of a long ssDNA can be discerned from the real-time observation (see video in Supporting Information), indicating that the ssDNA was only tethered by one end of the strand.

The nonspecific interaction between the DNA complex and the TPP sidewall surface is also considered to play an important role in assisting the DNAP docking. Since the RCA was performed without bias, the docked DNAPs were likely to be stalled at the TPPs mainly due to the nonspecific adhesion. As discussed previously, the slightly positively charged HfO_2_ surface could provide a Coulombic attraction to stabilize the polymerase–DNA complex for the docking process. Despite the lack of direct means to confirm the origin of the observed ssDNA, it is unlikely that they were synthesized elsewhere in the chamber and then drifted to the nanopores. On the contrary, two reasons support our conclusion that they were indeed synthesized by the docked DNAP. First, the polymerase–DNA complexes were only added to the *cis* chamber for docking. Hence, if the DNAP elsewhere in the *cis* chamber had synthesized ssDNA, the electrophoretic force in the *cis* chamber due to the negative bias (−50 mV) would drive the ssDNA away from the nanopore region. Second, if the ssDNA synthesized elsewhere could diffuse to the nanopore membrane and stuck to the surface during the RCA process without bias, they should be randomly distributed over the membrane surface. However, the observed ssDNA molecules were only found inside the array region.

In this demonstration, the initial number of open TPPs in the array was 23, while the remaining two TTPs were closed after the ALD coating. Docking of the polymerase–DNA complex was observed with high certainty for 7 of the 23 open TPPs, indicated by drastically decreased fluorescent intensity caused by the blockage of the Ca^2+^ ion flux. After the RCA reaction, 3 of the 7 TPPs showed the fluorescence signal from the synthesized ssDNA. Of the 3, 1 elongated ssDNA was obvious, while the other 2 showed relatively weak fluorescent signals (Figure 5d). The low rate of success in synthesizing ssDNA by docked DNAP, i.e., 3 out of 25, is mainly due to too many large TPPs in the array. Optimization of the fabrication process to achieve better uniformity in pore size and obtain smaller pores is necessary.

## Conclusions

The docking dynamics of the polymerase–DNA complex on nanopore arrays has been investigated using both electrical and optical readout schemes. By combining truncated-pyramidal Si nanopores with conformal ALD coating of a HfO_2_ layer, sub-10 nm nanopores have been realized for probable docking of DNAP 5–8 nm in size. The electrical measurement data on a single pore show that the DNAP–template complex can temporally dock on a TPP of 8 nm opening size over several seconds with certain DNAP orientations, while the majority of events are translocations. The optical measurement on 5 × 5 pore arrays demonstrates that employing the Ca^2+^ indicator dye to monitor the Ca^2+^ flux is able to report DNAP docking events on multiple pores simultaneously without needing to label the analyte. Additionally, the activity of the docked DNAP is examined by performing *in situ* RCA and a synthesized ssDNA tethered to the TPP array is observed, which proves the retained activity of phi29 DNAP. Thus, this detection scheme shows the possibility to introduce motor enzymes for single-molecule sensing applications. From the application perspective, the docking experiment can be improved using nanopore arrays with a reliable sub-5 nm pore size to prevent DNAP translocation and an advanced optical system to attain a raised resolution for dynamic studies.

## Supporting Information Available

Schematic process flow for fabrication of HfO_2_ coated Si nanopore array; Fluidic cell design for optical observation; SEM images of specially designed pattern for cross-section evaluation; SEM and TEM images of blocked TPP after ALD coating; Correlation of the working DNAP with the position of nanopore array (PDF)
Real-time video of ssDNA synthesized by docked DNAP on the TPP array (MP4)
