# Supplementary material for: Docking and Activity of DNA Polymerase on Solid-State Nanopores
Source: ACS Sens. 2022 May 10;7(5):1476–83. doi: 10.1021/acssensors.2c00216 (PMC9150166; doi:10.1021/acssensors.2c00216)
Supplement: Supplementary file 1 — se2c00216_si_001.pdf [file se2c00216_si_001.pdf]

SUPPORTING INFORMATION

Docking and Activity of DNA Polymerase on Solid-State Nanopores

*Shiyu Li,[1] Shuangshuang Zeng,[1] Chenyu Wen,[1] Zhen Zhang,[1] Klas Hjort,[2] Shi-Li Zhang[1]**

[1]Department of Electrical Engineering, Division of Solid-State Electronics, Uppsala University, SE-751 03 Uppsala, Sweden

[2]Department of Material Science and Engineering , Division of Microsystem Technology, Uppsala University, SE-751 21 Uppsala, Sweden

* To whom correspondence should be addressed: shili.zhang@angstrom.uu.se

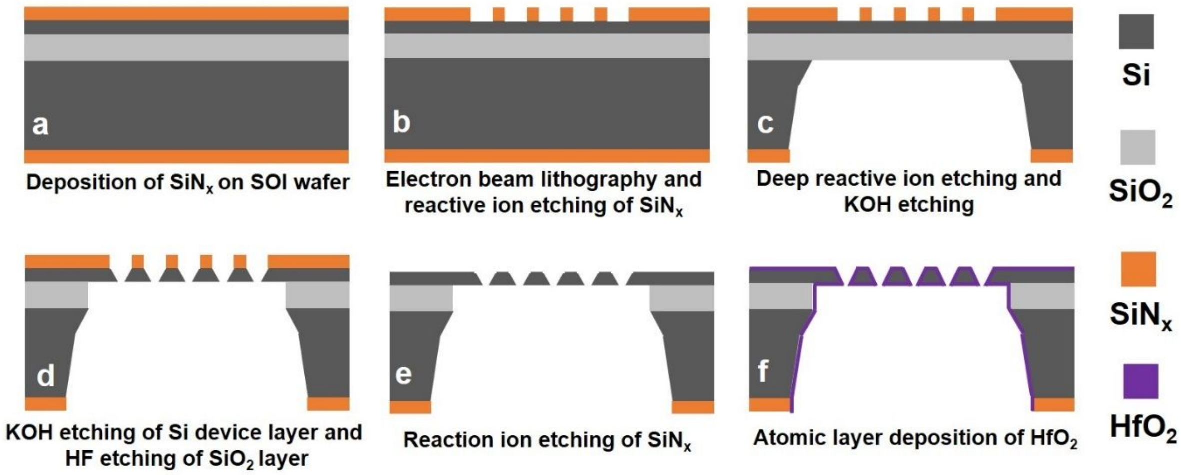

Figure S1. Process flow for fabrication of HfO_2 coated Si nanopore array: (a) Low-pressure vapor chemical deposition (LPCVD) of low-stress SiN_x on both sides of the SOI wafer; (b) EBL to form nanopores in the front-side SiN_x and photolithography to create a large window in the rear-side SiN_x being aligned to the front side nanopores; (c) Rear-side Si etching by means of deep reactive ion etching (DRIE) and wet KOH etching; (d) Transfer of the nanopore pattern from SiN_x to the Si layer on top of the BOX layer by a second KOH etching followed by removing the BOX layer in the large window using buffered hydrofluoric acid (BHF) etching; (e) Removal of SiN_x hardmask layer by RIE; (f) Atomic layer deposition (ALD) of HfO_2 to coat the entire surface of the structure including the Si nanopores.

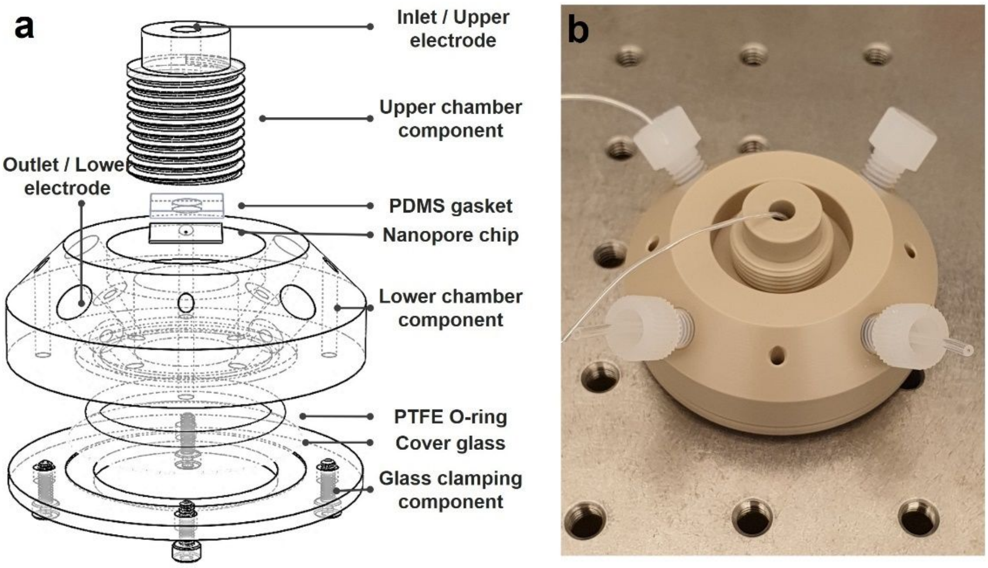

Figure S2. Fluidic cell for optical observation: (a) Schematic of the fluidic cell design for the optical observation; (b) Image of the assembled fluidic cell.

In order to obtain a good cross section of the TPPs, a specially designed pattern with ultrahigh pore density was prepared in the SiN_x mask layer, as shown in Figure S3. The arrangement of the pore arrays could ensure that the middle of a certain percentage of pore structure would be hit when slicing the wafer along its <110> direction. The slicing was then conducted through the pattern area with the bulk silicon substrate intact. After slicing, the chip was briefly etched in buffered HF to partly remove the BOX layer on the cross-section side, which will make the Si membrane layer at the edges suspended and ready for cross-sectional characterization.

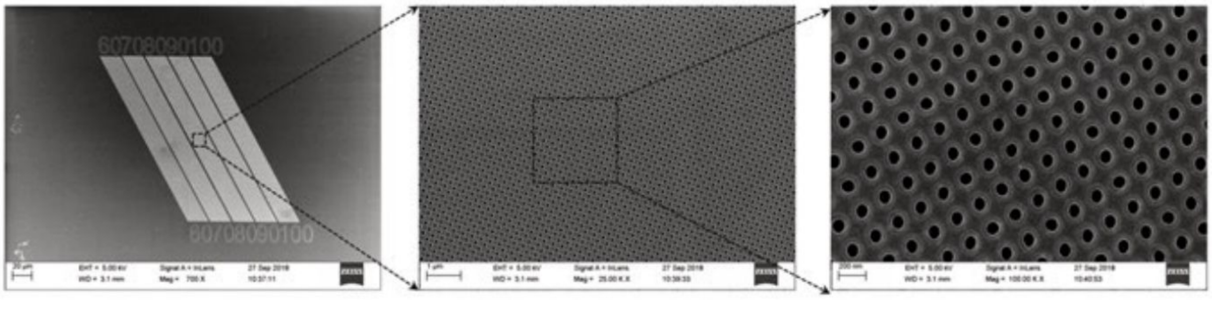

Figure S3. SEM images of a specially designed pattern with ultrahigh pore densities for cross-section evaluation.

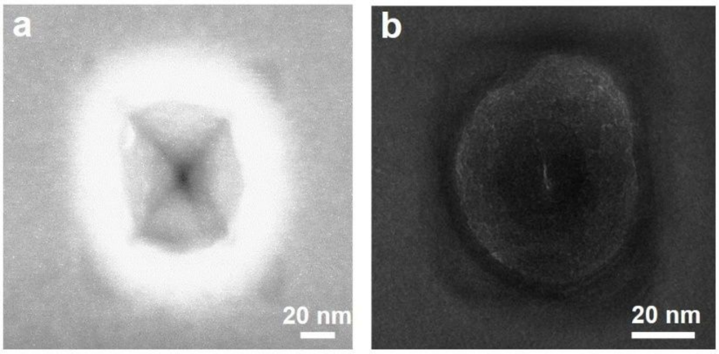

Figure S4. Blocked TPP after ALD coating: (a) SEM image of a blocked TPP after coating of HfO_2 to shrink the pore size. (b) TEM image of a blocked TPP pore after coating of HfO_2.

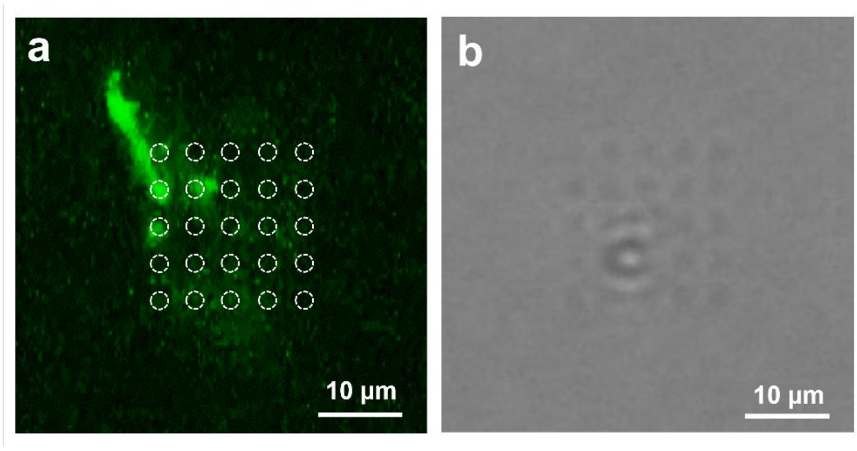

Figure S5. Correlation of the working DNAP with the position of nanopore array: (a) Fluorescence image of the observed ssDNA synthesized by the docked DNAP. The white dashed circles mark the nanopores. (b) Optical image of the nanopore array in the transmission channel. Due to the truncated-pyramidal structure of the nanopores and the interference of transmission light, a vague silhouette of the 5-by-5 nanopore array can be discerned.
